# The genus *Dettopsomyia* Lamb, 1914 (Diptera, Drosophilidae) from southern China

**DOI:** 10.3897/zookeys.1056.56996

**Published:** 2021-08-19

**Authors:** Ya-Lian Wang, Qiao Li, Masanori J. Toda, Jian-Jun Gao

**Affiliations:** 1 Yunnan Key Laboratory of Plant Reproductive Adaptation and Evolutionary Ecology, Yunnan University, Kunming, Yunnan 650091, China Southwest Forestry University Kunming China; 2 School of Forestry, Southwest Forestry University, Kunming, Yunnan 650224, China Yunnan University, Kunming Kunming China; 3 Hokkaido University Museum, Hokkaido University, Sapporo, Japan Hokkaido University Sapporo Japan; 4 Laboratory of Ecology & Evolutionary Biology, Yunnan University, Kunming, Yunnan 650091, China Yunnan University Kunming China

**Keywords:** *
Dettopsomyia
*, DNA barcoding, new species, *
Styloptera
*, Yunnan

## Abstract

The genus *Dettopsomyia* was established by Lamb in 1914 for a single species, *De.formosa* described therein. It contains 13 known species recorded from the Old World (the Oriental, Australasian, Palearctic and Afrotropical regions). In the present paper, five new species discovered from southern China are described as members of *Dettopsomyia*: *De.acutipenis* Wang & Gao, **sp. nov.**, *De.serripenis* Wang & Gao, **sp. nov.**, *De.discontinua* Wang & Gao, **sp. nov.**, *De.camelonota* Wang, Li & Gao, **sp. nov.** and *De.paranigrovittata* Wang, Li & Gao, **sp. nov.** The new species were delimitated, based on not only morphological characters but also molecular data.

## Introduction

The genus *Dettopsomyia* was established by Lamb (1914) for *De.formosa* described therein as the type species. Since then, a number of species have been added as new members to this genus or transferred from other genera by some authors, bringing the total number of known species in *Dettopsomyia* to 13. Duda (1926) described two Indonesian species, *Dettopsomyiajacobsoni* and *De.acrostichalis*, and transferred *Drosophilapictipes* de Meijere, 1911 from Indonesia and the Philippines into *Dettopsomyia*, together with *Pictostylopterapreciosa* (de Meijere, 1911) from Indonesia and *Stylopterafruhstorferi* Duda, 1924 from Vietnam. Wheeler (1951) transferred the Australasian species *Drosophilanigrovittata* Malloch, 1924 to *Dettopsomyia*. In addition, four more species, *Dettopsomyiaequscauda* Takada & Momma, 1975 from Malaysia, *De.philippina* Takada, 1976 from the Philippines, *De.alba* Carson & Okada in Okada (1982) from Papua New Guinea and *De.woodruffi* Takada in Takada et al. (1990) from Kenya, have been described as new members of this genus, and two other species, *Mycodrosophilabombax* Burla, 1954 from Ivory Coast and Uganda and *Stylopterarepletoides* Carson & Okada, 1980 from Papua New Guinea, have been transferred to *Dettopsomyia* by Tsacas (1980) and Okada (1982), respectively. Currently, a total of 13 species is assigned to this genus and are mainly distributed in the Old World tropics, i.e., the Oriental, Australasian and Afrotropical regions (Toda 2020).

From the early days of this taxonomic history, the status of the genus *Dettopsomyia* has been argued, especially in relation to the genus *Styloptera* Duda. Duda (1924) established the genus *Styloptera* for two new species, *S.formosae* Duda, 1924 and *S.fruhstorferi* Duda, 1924, and one known species, *S.pictipes* (de Meijere, 1911), transferred from the genus *Drosophila* Fallén, and the genus *Pictostyloptera* for *Drosophilapreciosa* de Meijere, 1911. But later, Duda (1926) regarded both genera *Styloptera* and *Pictostyloptera* as synonymous with *Dettopsomyia* by finding intermediate morphologies between these genera in a study of *Dettopsomyiaformosa* (the type species). Wheeler and Takada (1964) resurrected the genus *Styloptera* by choosing *S.formosae* as the type species because of its distinct morphology, but leaving *De.fruhstorferi* and *De.pictipes* in *Dettopsomyia*. Then, nine new species were added to the genus *Styloptera* by Okada and Carson (1980, 1983), Okada (1982) and Bock (1982), currently resulting in a total of ten species (Toda 2020). Wheeler and Takada (1964) and Bock (1982) gave the morphological diagnoses mostly on the head/thorax (color pattern and chaetotaxy) and the wing (color pattern and venation) for *Dettopsomyia* and *Styloptera*. However, some characters listed in the diagnoses were not contradictory between these two genera, but partially overlapping between them. Okada (1982) compared the morphology of the two genera in a revisional study covering 12 *Dettopsomyia* and eight *Styloptera* species. He listed some characters common to the two genera, e.g., wing costal lappet more or less developed and black, dorsocentral setae usually in three pairs, and frons and thorax usually ornamented, indicating close relationships between them. On the other hand, he proposed 13 characters (Table 1) as being diagnostic to distinguish between these two genera, at least for a few ‘representative’ species of them. However, none of these characters is applicable to most of the component species of either genus (Okada 1982). Bock (1982) argued that these two genera are closely related but most of their component species are very poorly known, and proposed that a complete revision of the two genera is necessary.

**Table 1.** Diagnostic characters used to distinguish between the genera *Dettopsomyia* and *Styloptera* (adapted from Okada 1982).

* Dettopsomyia *		* Styloptera *	
Code	State	Code	State
A	Eye much oblique to the body axis.	a	Eye nearly rectangular to the body axis.
B	Ocellar setae inserted inside triangle made by ocelli.	b	Ocellar setae inserted outside triangle made by ocelli.
C	Anterior reclinate orbital minute.	c	Anterior reclinate orbital >> 1/3 as long as proclinate.
D	Cheek not very broad, ~ 1/3 as broad as greatest diameter of eye.	d	Cheek very broad, ~ 2/3 as broad as greatest diameter of eye.
E	Costal lappet large.	e	Costal lappet moderate.
F	C-index < 1.0.	f	C-index > 1.0.
G	R_2+3_ strongly curved to costa apically.	g	R_2+3_ straight or merely gently curved to costa.
H	R_4+5_ and M_1_ divergent distally.	h	R_4+5_ and M_1_ parallel.
I	Acrostichal bristles present.	i	Acrostichal bristles absent.
J	Tibia ringed.	j	Tibia not ringed.
K	Wing spotted.	k	Wing not spotted.
L	Acrostichal setulae in 2 rows.	l	Acrostichal setulae in 4 or 6 rows.
M	C3-fringe > 1/2.	m	C3-fringe < 1/2.

As Bock (1982) pointed out, the two genera *Dettopsomyia* and *Styloptera* are still less explored, making a full-scale revision of their phylogeny and taxonomy difficult. In the present paper, we describe five new species of *Dettopsomyia* discovered from China, and briefly address the ambiguity of *Dettopsomyia* and *Styloptera* in the systematics of the subfamily Drosophilinae.

## Materials and methods

### Specimens

Taxon sampling for morphological examination and DNA barcoding is shown in Table 2. The specimens were mostly captured by net sweeping above herbs in open forest, or at forest edge. Specimens were preserved in 70% (for morphological examination) or 100% ethanol (for DNA sequencing).

**Table 2.** Summary of *Dettopsomyia* species and specimens examined in this study. Voucher numbers in bold indicate holotype specimens; gender of each specimen is given in parentheses, and GenBank accession numbers of *COI* sequences in brackets.

Species	Collection site	Collection date	Voucher #
*De.acutipenis* sp. nov.	Xishuangbanna Tropical Botanical Garden, Mengla, Xishuangbanna, Yunnan, China	19.iii.2006	#00138 (♀) [MZ645108], #**00151** (♂)
		18.iv.2007	#00380 (♀) [MZ645110], #00381 (♀) [MZ645111], #00382–386 (5♀)
		16.iv.2007	#00387–389 (3♀)
*De.serripenis* sp. nov.	Xishuangbanna Tropical Botanical Garden, Mengla, Xishuangbanna, Yunnan, China	19.iii.2006	#**00152** (♂), #00155 (♂), #00156 (♀) [MZ645109], #00157 (♂), #00158 (♀)
		24.iii.2006	#00153 (♂)
		25.iii.2006	#00154 (♀)
*De.discontinua* sp. nov.	Baihualing, Baoshan, Yunnan, China	4.viii.2012	#01139 (♂) [MZ645112], #01140 (♂) [MZ645113], #01141 (♂), #01142 (♂), #01143 (♀) [MZ645114], #01144 (♂) [MZ645115]
	From rearings of *R.decursiva* infructescences collected from Baihualing, Baoshan, Yunnan, China	23.ix.2012	#01167–169 (3♀) [MZ645117–119], #01172–174 (3♂) [MZ645120–122]
	Banpo, Yixiang, Simao, Pu’er, Yunnan, China	2.x.2012	#01584 (♂) [MZ645136], #**01585** (♂) [MZ645137]
*De.camelonota* sp. nov.	Banpo, Yixiang, Simao, Pu’er, Yunnan, China	25.x.2012	#**01607** (♂) [MZ645138], #01608 (♀) [MZ645139]
*De.paranigrovittata* sp. nov.	Baihualing, Longyang, Baoshan, Yunnan, China	3.viii.2012	#**01145** (♂) [MZ645116]
	From rearings of host infructescences collected from Baihualing, Baoshan, Yunnan, China	23.ix.2012	#01579 (♂) [MZ645131]
*De.nigrovittata* (Malloch, 1924)	Dinghushan Nature Reserve, Zhaoqing, Guangdong, China (by net sweeping above herbs)	13.iv.2008	#00132 (♂) [MZ645104], #00135–137 (3♀) MZ645105–107]
		23–26.iii.2009	#01582 (♀) [MZ645134], #01583 (♂) [MZ645135]
	From rearings of infructescences of *Rhaphidophoradecursiva* collected from Baihualing, Baoshan, Yunnan, China	5.ix.2012	#01177 (♀) [MZ645123], #01178 (♂) [MZ645124], #01179 (♂) [MZ645125], #01180 (♀) [MZ645126], #01182–184 (3♀) [MZ645127–129]
		23.ix.2012	#01578 (♀) [MZ645130], #01580 (♂) [MZ645132], #01581 (♀) [MZ645133], #01721 (♀) [MZ645141]
	Haoping Station, Taibaishan National Nature Reserve, Shaanxi, China (by net sweeping above herbs along waterside)	16.viii.2013	#01680 [MZ645140]

The examined specimens are deposited in the following institutes:

**KIZ**Kunming Natural History Museum of Zoology, Kunming Institute of Zoology, Chinese Academy of Sciences, Kunming, China;

**SEHU** Systematic Entomology, the Hokkaido University Museum, Hokkaido University, Sapporo, Japan.

### Species delimitation and description

As pointed out by Okada (1982) and Bock (1982), it is difficult to definitely determine the generic assignment of the studied specimens to *Dettopsomyia* Lamb, 1914 or *Styloptera* Duda, 1924, based on morphological characters, for the present. As we address this issue in Discussion, it needs a systematic revision for these genera based on a full-scale molecular phylogenetic analysis of the subfamily Drosophilinae and ancestral state reconstruction of the morphology by character-mapping on the inferred tree. In the present study, therefore, we provisionally classify all studied specimens into *Dettopsomyia* according to the principle of priority (ICZN), since there is a possibility that the two genera are synonymized in future systematic studies. Then, they were classified into morpho-species referring to Okada’s (1982) 13 characters (Table 1): the character states (referred to as CS-code in descriptions of species) for all the known and putatively new species of *Dettopsomyia* are summarized in Table 3. The morpho-species were further examined for other external morphology and detailed structures of dissected phallic and periphallic organs by the same methods as in Li et al. (2014). For each morpho-species, at least one representative specimen was selected for mitochondrial *COI* DNA sequencing. We followed Li et al. (2014) and Yang et al. (2017) for extraction of DNA, PCR and sequencing, using Folmer et al. (1994) primer pair LCO1490 (5’- GGTCAACAAATCATAAAGATATTGG -3’) and HCO2198 (5’- TAAACTTCAGGGTGACCAAAAAATCA -3’). The sequences were edited in the SeqMan module of the DNAStar package (DNAStar Inc. 1996), and aligned in MEGA7 (Kumar et al. 2016). We performed tree- and distance-based DNA barcoding, with a neighbor-joining (NJ) tree constructed in MEGA7 with K2P distances (i.e., the Kimura 2-parameter distances) and comparison of the maximum intraspecific and the minimum interspecific p-distances. The morpho-species were then reconsidered by integrating information from the morphology and DNA barcode data.

McAlpine (1981) was followed for the morphological terminology, and Zhang and Toda (1992) for the definitions of measurements and indices.

**Table 3.** A character-state matrix of Okada’s (1982) 13 characters for all *Dettopsomyia* species.

**Species**	**Characters**													Reference*
	**1**	**2**	**3**	**4**	**5**	**6**	**7**	**8**	**9**	**10**	**11**	**12**	**13**	
*De.formosa* Lamb, 1914	A	B	C	D	E	F	G	H	i	J	K	l	M	1)
*De.jacobsoni* Duda, 1926	A	B	C	D	E	F	G	H	I	J	K	L	M	1)
*De.preciosa* (de Meijere, 1911)	A	B	C	D	e	f	G	H	I	J	K^†^	L	m	1)
*De.fruhstorferi* (Duda, 1924)	–	–	–	–	–	f	G	–	i	J	k	–	–	1)
*De.pictipes* (de Meijere, 1911)	A	B	C	D	e	f	G	H	I	J	k	L	m	1)
*De.repletoides* (Carson & Okada, 1980)	a	b	c	d	E	f	G	H	i	J	k	L	m	1)
*De.alba* Carson & Okada, 1982	a	b	c	d	E	f	G	H	i	j	k	L	m	1)
*De.acrostichalis* Duda, 1926	–	–	–	–	–	–	G	h	I	–	k	–	–	1)
*De.nigrovittata* (Malloch, 1924)	a	b	c	d	E	F	G	h	i	J	k	l	M	1)
*De.philippina* Takada, 1976	a	b	C	d	E	f	g	h	i	J	k	l	m	1)
*De.equscauda* Takada & Momma, 1975	A	b	c	d	e	f	G	H	i	J	k	L	m	1)
*De.bombax* (Burla, 1954)	–	–	C	D	E	f	G	h	i	j	k	l	m	1)
*De.woodruffi* Takada, 1990	A	b	–	D	E	–	G	h	i	J	k	l	M	2)
*De.acutipenis* sp. nov.	A	b	?	?	e	f	G	H	i	J	k	l	M	3)
*De.serripenis* sp. nov.	A	b	C	D	?	?	G	H	i	J	K	?	M	3)
*De.discontinua* sp. nov.	A	b	C	D	?	F	G	H	i	J	K	L	M	3)
*De.camelonota* sp. nov.	A	B	C	D	?	F	G	h	i	J	K	?	m	3)
*De.paranigrovittata* sp. nov.	a	b	c	D	?	f	g	h	i	J	k	l	M	3)

The symbols “-” and “?” in the table indicate missing data and ambiguous state, respectively. * Reference: 1) Okada (1982), 2) Takada et al. (1990), and 3) the present study. ^†^ Revised according to de Meijere (1911: fig. 49).

## Results

### Species delimitation

**Figure 1.**
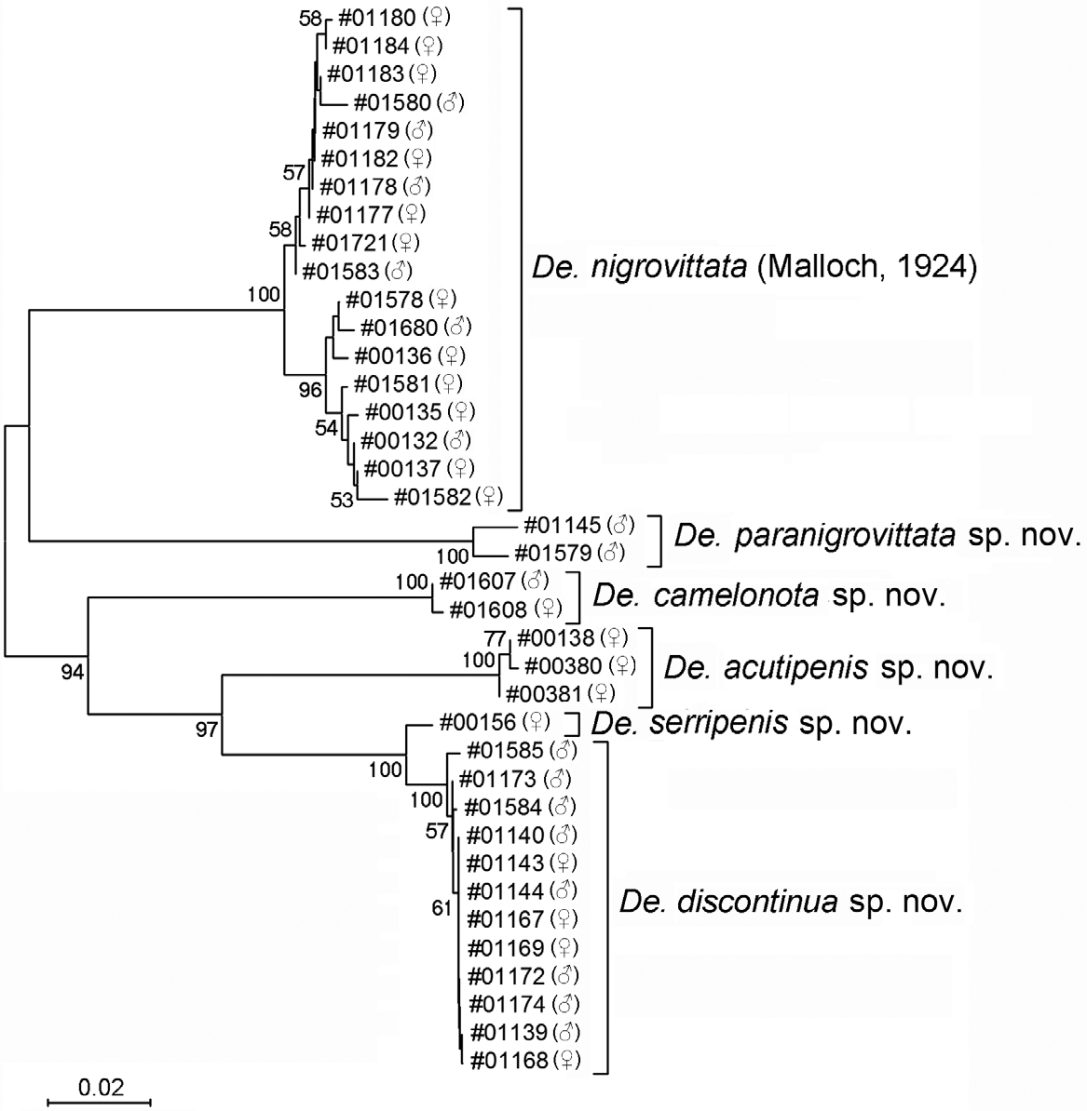
An un-rooted, neighbor-joining tree built with DNA barcodes (mitochondrial *COI* sequences) of six *Dettopsomyia* species. Label of each operational taxonomic unit (OTU) is given in the format of “voucher number (sex)”. Node confidences (i.e., bootstrap percentages from 1000 replicates) ≥ 50% are shown.

The specimens were assigned into six morpho-species (one known and five new) of the genus *Dettopsomyia*. The alignment of the 38 barcodes spans (658 nucleotide sites in length) included 169 variable sites, among which 156 were parsimony informative. Fig. 1 shows the NJ tree built with the barcodes (GenBank accession numbers: MZ645104–MZ645141). The tree lends strong supports to the monophyly of each of the morpho-species with BP (bootstrap percentage) = 100, except *De.serripenis* sp. nov. for which only one barcode was determined. The minimum and maximum K2P distances between and within the morpho-species are shown in Table 4. All the minimum interspecific K2P distances (≥ 0.0924), except for that between *De.serripenis* sp. nov. and *De.discontinua* sp. nov. (0.0132), were substantially larger than the maximum intraspecific distances (≤0.0391). *Dettopsomyiaserripenis* sp. nov. formed a highly supported clade (BP = 100) with a compact cluster (K2P ≤ 0.0048, BP = 100) of 12 barcodes of *De.discontinua* sp. nov. (Fig. 1, Table 4). However, the former is readily distinguished from the latter in the morphology of both male and female (see the morphological diagnosis defined below for *De.discontinua* sp. nov.). On the other hand, the largest intraspecific distance (0.0391) was observed within the morpho-species *De.nigrovittata* (Table 4). Although the 18 barcode sequences of this species formed a monophyletic cluster (BP = 100), they were split into two subclusters with BPs = 58 and 96 (Fig. 1). However, no significant differentiation attributable to this subdivision was detected in either morphology (see Taxonomic account), habitat or geographical distribution (Table 2) between the two subclusters. We therefore regarded all these 18 specimens as of the same species (i.e., *De.nigrovittata*). Similarly, we identified two specimens of which K2P distance (0.0152) slightly exceeded the least interspecific distance (0.0132) (Table 4) as *De.paranigrovittata* sp. nov. based on the morphology.

**Table 4.** Minimum and maximum of intra- and interspecific K2P distances of six *Dettopsomyia* species.

Species	*n* ^a^	Intraspecific distance		Interspecific distances^b^					
		Minimum	Maximum	1	2	3	4	5	6
*De.nigrovittata* (Malloch, 1924)	18	0.0000	0.0391		0.1355	0.1330	0.1499	0.1380	0.1420
*De.paranigrovittata* sp. nov.	2	0.0152	0.0152	0.1741		0.1784	0.1946	0.1776	0.1708
*De.camelonota* sp. nov.	2	0.0000	0.0000	0.1621	0.1897		0.1435	0.1372	0.1320
*De.acutipenis* sp. nov.	3	0.0000	0.0017	0.1829	0.2069	0.1489		0.0978	0.0924
*De.serripenis* sp. nov.	1	n/a	n/a	0.1484	0.1780	0.1445	0.1046		0.0132
*De.discontinua* sp. nov.	12	0.0000	0.0048	0.1756	0.1847	0.1434	0.1035	0.0164	

^a^ Number of sequences; ^b^ Maximum distances below diagonal, and minimum distances above diagonal.

### Taxonomic account

#### Genus Dettopsomyia

Taxon classificationAnimaliaDipteraDrosophilidae

Lamb, 1914

C5F94EE4-4219-562D-BB82-7D65877E3E13

Dettopsomyia Lamb, 1914: 349; Wheeler & Takada 1964: 210; Bock 1982: 42; Okada 1982: 270; Bächli et al. 2004: 119. Type species: Dettopsomyiaformosa Lamb, 1914.
Pictostyloptera Duda, 1924: 192. Syn. Duda 1926: 61. Type species: Drosophilapreciosa de Meijere, 1911.

##### Included species.

*acrostichalis* Duda, 1926; *alba* Carson & Okada in Okada (1982); *bombax* (Burla, 1954); *equscauda* Takada & Momma, 1975; *formosa* Lamb, 1914; *fruhstorferi* (Duda, 1924); *jacobsoni* Duda,1926; *nigrovittata* (Malloch, 1924); *philippina* Takada, 1976; *pictipes* (de Meijere, 1911); *preciosa* (de Meijere, 1911); *repletoides* (Carson & Okada, 1980); *woodruffi* Takada in Takada et al. (1990); *acutipenis* Wang & Gao, sp. nov.; *camelonota* Wang, Li & Gao sp. nov.; *discontinua* Wang & Gao, sp. nov.; *paranigrovittata* Wang, Li & Gao, sp. nov.; and *serripenis* Wang & Gao, sp. nov.

##### Geographical distribution.

**Figure 2.**
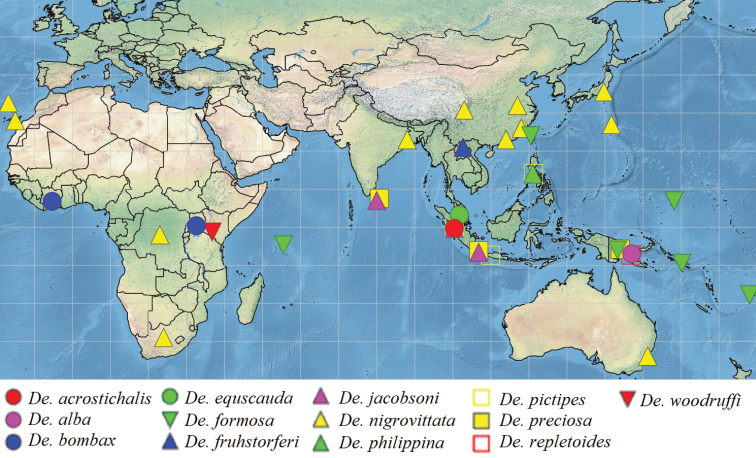
Geographical distribution of the known species in the genus *Dettopsomyia*.

**Figure 3.**
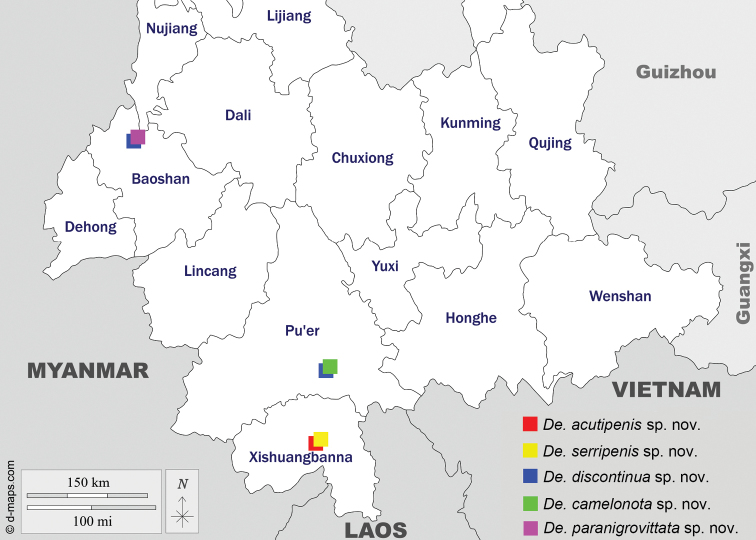
Geographical distribution of five newly described species in the genus *Dettopsomyia*.

Collection records of all the known species from the world are plotted in a map (Fig. 2) using Simplemappr (http://www.simplemappr.net/), and collection sites of the five new species plotted in a separate, online map (Fig. 3) from https://d-maps.com.

### Key to Oriental species of *Dettopsomyia*

In this key, some figures published by Lamb (1914), Duda (1924, 1926), Okada (1956, 1982), Hardy (1965), and Takada (1976) are cited.

**Table:** 

1	Wing spotted (Figs 5C, 6C, 7C; Lamb 1914: fig. 33; Duda 1924: fig. 39; Duda 1926: fig. 3; Hardy 1965: fig. 20b).	**2**
–	Wing not spotted (Figs 4C, 8C; Duda 1924: fig. 40).	**7**
2	Acrostichal bristles present (Okada 1982: fig. 1B, C).	**3**
–	Acrostichal bristles absent (Figs 5–7B; Okada 1982: fig. 1A).	**4**
3	Wing with two black spots along costa (Duda 1924: fig. 39).	***De.preciosa* (de Meijere)**
–	Wing with four black spots along costa (Duda 1926: fig. 3).	***De* . *jacobsoni* Duda**
4	Wing with approximately 24 pale spots; R_4+5_ and M_1_ parallel (Fig. 7C).	***De.camelonota* Wang, Li & Gao, sp. nov.**
–	Wing with 14–17 pale spots; R_4+5_ and M_1_ divergent distally (Figs 5C, 6C; Lamb 1914: fig. 33; Hardy 1965: fig. 20b).	**5**
5	Ocellar setae inserted inside triangle made by ocelli (Okada 1982) fig. 1A); epandrial, ventral lobe elongated (Okada 1982: fig. 2A).	***De.formosa* Lamb**
–	Ocellar setae inserted outside triangle made by ocelli (Figs 5B, 6B); epandrial, ventral lobe short (Fig. 5F, G) or absent (Fig. 6F, G).	**6**
6	Cercus somewhat pointed but not protruded caudoventrally (Fig. 5F); surstylus with 14 or 15 prensisetae arranged in V-shape (Fig. 5F, G); marginal peg-like ovisensilla in continuous row (Fig. 5J, K).	***De.serripenis* Wang & Gao, sp. nov.**
–	Cercus caudoventrally protruded ventrad like finger (Fig. 6F); surstylus with approximately 11 prensisetae on distal margin and 9 or 10 ones on medial portion of outer surface, arranged together nearly in circle (Fig. 6F, G); row of marginal peg-like ovisensilla interrupted around subterminal, long, trichoid seta (Fig. 6J, K).	***De.discontinua* Wang & Gao, sp. nov.**
7	R_2+3_ nearly straight (Fig. 8C; Takada 1976: fig. 1c).	**8**
–	R_2+3_ more or less curved to costa apically (Fig. 4C).	**9**
8	Scutum and scutellum blackish brown to black (Fig. 8B).	***De.paranigrovittata* Wang, Li & Gao, sp. nov.**
–	Scutum and scutellum with brownish, longitudinal stripes (Takada 1976: fig. 1a).	***De.philippina* Takada**
9	Acrostichal bristles present.	**10**
–	Acrostichal bristles absent (Figs 4B, 8B).	**11**
10	R_4+5_ and M_1_ divergent distally (Duda 1924: fig. 40).	***De.pictipes* (de Meijere)**
–	R_4+5_ and M_1_ parallel	***De.acrosticholis* Duda**
11	C-index < 1.0 (Okada 1956: fig. 31).	***De.nigrovittata* (Malloch)**
–	C-index > 1.0 (Fig. 4A).	**12**
12	Scutum with 10 dark stripes	***De.fruhstorferi* (Duda)**
–	Scutum with < 10 dark stripes (Fig. 4B), or with complex dark marks.	**13**
13	Acrostichal setulae in 2 rows.	***De.equscauda* Takada & Momma**
–	Acrostichal setulae in 4 rows (Fig. 4B).	***De.acutipenis* Wang & Gao, sp. nov.**

### Descriptions of species

#### Dettopsomyia acutipenis

Taxon classificationAnimaliaDipteraDrosophilidae

Wang & Gao sp. nov.

BE432337-F1D4-522D-9D2A-6186A1F6C519

http://zoobank.org/46AA8D2F-D9A1-43BB-A251-524BB1E60157

Figure 4

##### Material.

***Holotype*** ♂ (#00151), Yunnan: Xishuangbanna Tropical Botanical Garden, the Chinese Academy of Sciences, Menglun, Mengla, Xishuangbanna, ca. 570 m (21.92°N, 101.28°E), 19.iii.2006, *ex* flower of *Zinger* sp. (M.J. Toda) (KIZ). ***Paratypes*** China: 7♀ (#00380–386), the data same as holotype except for 18.vi.2007 (J.J. Gao); 3♀ (#00387–389), the data same as holotype except for 16.vi.2007 (J.J. Gao) (KIZ).

##### Diagnosis.

This species differs in CS-code (Ab??efGHiJklM) from all the remaining congeneric species, except for *De.fruhstorferi* (?????fG?iJk?? according to Okada, 1982), which is, however, distinguished from the new species by the number of dark, longitudinal stripes on scutum: six in the new species, but ten in *De.fruhstorferi*.

##### Description.

**Figure 4.**
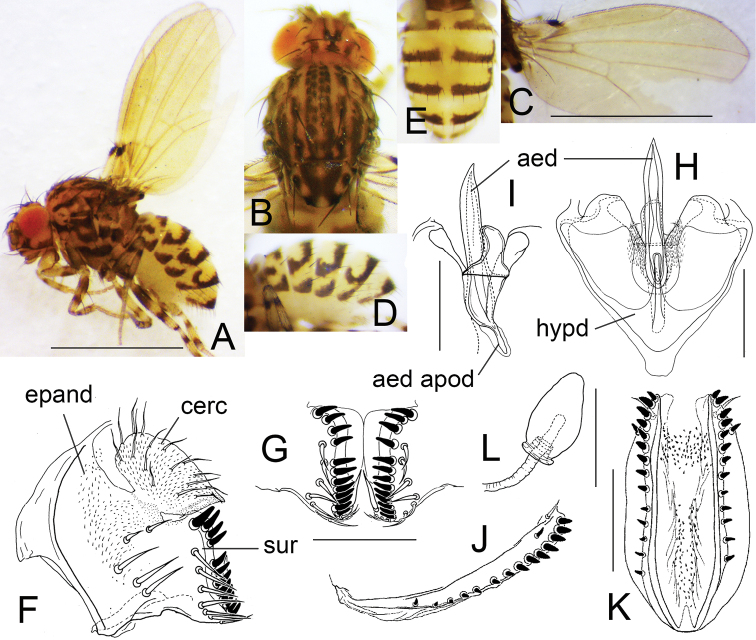
*Dettopsomyiaacutipenis* Wang & Gao, sp. nov. (**A–I** #00151, **J–L** paratype #00380) **A** left lateral habitus **B** head and thorax (dorsal view) **C** wing (right, dorsal view) **D** abdomen (lateral view) **E** abdomen (dorsal view) **F** periphallic organs (posterolateral view) **G** surstylus **H** phallic organs (ventral view) **I** aedeagus (dorsolateral view) **J** oviscapt (lateral view) **K** oviscapt (ventral view) **L** spermatheca. Abbreviations: aed = aedeagus, aed apod = aedeagal apodeme, cerc = cercus, epand = epandrium, hypd = hypandrium, sur = surstylus. Scale bars: 1.0 mm (photograph) or 0.1 mm (line drawing).

(♂, ♀). ***Head*** (Fig. 4A, B): Eye red, much oblique to body axis, with dense interfacetal setulae. Ocellar triangle matte black. Ocellar setae located outside triangle made by ocelli. Fronto-orbital plate anteriorly with a black spot at the base of proclinate and anterior reclinate orbital setae. Frons grayish yellow, with pale brown stripes. Face yellow; carina large, ventrally with blackish brown, T-shaped spot. Clypeus black. Gena yellowish brown, with dark stripe along ventral margin. Palpus yellow. Pedicel (2^nd^ antennal segment) yellow, laterally with black spot; 1^st^ flagellomere (3^rd^ antennal segment) grayish yellow.

***Thorax*** (Fig. 4A, B): Scutum, scutellum, and thoracic pleura yellowish brown; scutellum apically white. Scutum with six blackish brown stripes; median pair close to each other. Scutellum medially with X-shaped, dark marking posteriorly covering the bases of apical scutellar setae, laterally with dark spots at the bases of basal scutellar setae. Thoracic pleura with three blackish, longitudinal stripes. Acrostichal long setae absent; acrostichal setulae in four rows. Dorsocentral setae three pairs. Basal scutellar setae slightly divergent; apical scutellar setae cruciate.

***Wing*** (Fig. 4C): Wing pale yellow. Veins yellowish brown. Costal lappet black, moderate in size. R_2+3_ curved to costa apically; R_4+5_ and M_1_ slightly diverged from each other distally. Haltere pale yellow, ventrally with small grayish patch; stalk slightly grayish.

***Legs*** (Fig. 4A): Legs yellow: femora and tibiae ringed.

***Abdomen*** (Fig. 4D, E): Tergites yellow, posteriorly with narrow, blackish brown, dorsomedially interrupted bands, which bend forward laterally, and with an isolated black spot on lateral margin.

***Male terminalia*** (Fig. 4F–I): Epandrium pubescent except for anterior margin and ventral portion, with five setae per side on ventral portion and triangular, distally somewhat roundish apodeme on antero-dorsal to -sublateral margin, slightly protruded anteriad at anteroventral corner. Surstylus broadly fused to epandrium; distal margin with a row of 12 or 13 peg-like, apically pointed prensisetae, which are more loosely arranged dorsally; outer surface with six or seven long, trichoid setae on subdorsal to ventral portion; caudoventral apex with a few short, trichoid setae. Cercus oblong, wider than 1/2 length, partially fused to epandrium, caudoventrally pointed, pubescent except for anteroventral margin, with approximately 17 setae. Hypandrium somewhat triangular, with a pair of minute setae (paramedian setae?), caudomedially deeply notched and revolute along inner edges, with large patches of pubescence on the revolute parts. Aedeagus apically acute, slightly curved dorsad, basally with recurved, dorsal flap; aedeagal guide broadly fused to revolute portion of hypandrium; apodeme fused to aedeagus, rod-like, < 1/3 length of aedeagus.

***Female terminalia*** (Fig. 4J–L): Oviscapt with single lateral and 13 or 14 marginal, apically more or less pointed, peg-like ovisensilla, and one subterminal, trichoid seta; anteroventral bridge short. Spermatheca somewhat fusiform, basally ridged; introvert ca. 1/4 height of outer capsule.

***Measurements***: BL (straight distance from anterior edge of pedicel to tip of abdomen) = 1.69 mm in holotype (range in 9♀ paratypes: 1.42–2.18 mm); ThL (distance from anterior notal margin to apex of scutellum) = 0.71 (0.62–0.74) mm; WL (distance from humeral cross vein to wing apex) = 1.46 (1.40–1.60) mm; WW (maximum wing width) = 0.73 (0.67–0.86) mm.

***Indices***: arb (dorsal branches/ventral branches of arista) = 3/2 in holotype (range in 9♀, or less if noted, paratypes: 3–4/2), FW/HW (frontal width/head width) = 0.59 (0.57–0.61), ch/o (maximum width of gena/maximum diameter of eye) = 0.42 (0.41–0.51), prorb (proclinate orbital seta/posterior reclinate orbital seta in length) = 0.70 (0.53–0.70), rcorb (anterior reclinate orbital seta/posterior reclinate orbital seta in length) = 0.33 (0.32–0.47), vb (subvibrissal seta/vibrissa in length) = 0.44 (0.24–0.47), dc_1_l (1^st^ dorsocentral seta/3^rd^ dorsocentral seta in length) = 0.91 (0.88–0.95), dc_2_l (2^nd^ dorsocentral seta/3^rd^ dorsocentral seta in length) = 0.78 (0.77–0.92), sctl (basal scutellar seta/apical scutellar seta in length) = (n/a)/(0.96–1.06), sterno (anterior katepisternal seta/posterior katepisternal seta in length) = 0.39 (0.39–0.56), orbito (distance between proclinate and posterior reclinate orbital setae/distance between inner vertical and posterior reclinate orbital setae) = 0.56 (0.40–0.61), dc_1_p (distance between ipsilateral 1^st^ and 2^nd^ dorsocentral setae/distance between 2^nd^ dorsocentral setae) = 0.76 (0.70–0.79), dc_2_p (distance between ipsilateral 2^nd^ and 3^rd^ dorsocentral setae/distance between 2^nd^ dorsocentral setae) = 0.79 (0.69–0.80), sctlp (distance between ipsilateral scutellar setae/distance between apical scutellar setae) = 0.94 (0.93–1.04), C (2^nd^ costal section between subcostal break and R_2+3_/3^rd^ costal section between R_2+3_ and R_4+5_) = 1.31 (1.17–1.51), 4c (3^rd^ costal section between R_2+3_ and R_4+5_/M_1_ between r-m and dm-cu) = 2.29 (1.72–2.54), 4v (M_1_ between dm-cu and wing margin/M_1_ between r-m and dm-cu) = 3.21 (2.40–3.36), 5× (CuA_1_ between dm-cu and wing margin/dm-cu between M_1_ and CuA_1_) = 3.82 (2.97–3.85), ac (3^rd^ costal section between R_2+3_ and R_4+5_/distance between distal ends of R_4+5_ and M_1_) = 2.92 (2.84–3.33), M (CuA_1_ between dm-cu and wing margin/M_1_ between r-m and dm-cu) = 1.43 (1.10–1.48), C3F (length of heavy setation in 3^rd^ costal section/length of 3^rd^ costal section) = 0.53 (0.50–0.60).

##### Distribution.

China (Yunnan).

##### Relationships.

This species closely resembles *De.repletoides* (CS-code = abcdEfGHiJkLm, Okada 1982) in the structures of male terminalia Carson and Okada 1980: fig. 3).

##### Etymology.

Referring to the apically more or less acute (*acuti*-) aedeagus (*penis*).

#### Dettopsomyia serripenis

Taxon classificationAnimaliaDipteraDrosophilidae

Wang & Gao sp. nov.

56930A24-2869-5297-B43C-71D7A04D840E

http://zoobank.org/7B6F20BB-1217-4333-9A45-F11C20F50D4A

Figure 5

##### Material.

***Holotype***: ♂ (#00152), Xishuangbanna Tropical Botanical Garden, the Chinese Academy of Sciences, Menglun, Mengla, Xishuangbanna, Yunnan, China, ca. 570 m (21.92°N, 101.28°E), 19.iii.2006, *ex*Zingerberaceae flower (M.J. Toda) (KIZ). ***Paratypes***: China: 1♂ (#00153), same data except for 24.iii.2006, *ex Alocasiaodora*; 1♀ (#00154), same data except for 25.iii.2006; 2♂, 2♀ (#00155–158), same data except for habitat (host plant unknown) (M.J. Toda) (KIZ, SEHU).

##### Diagnosis.

**Figure 5.**
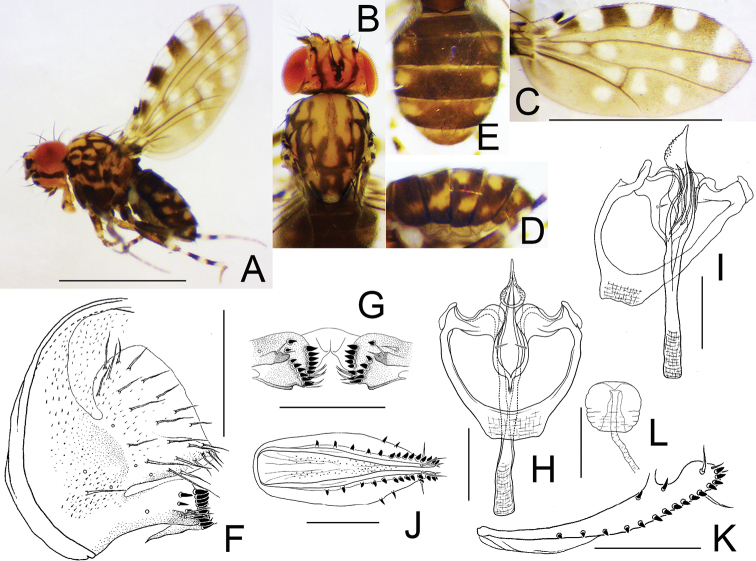
*Dettopsomyiaserripenis* Wang & Gao, sp. nov. (**A–I** #00152, **J–L** paratype #00156) **A** left lateral habitus **B** head and thorax (dorsal view) **C** wing (right, dorsal view) **D** abdomen (lateral view) **E** abdomen (dorsal view) **F** periphallic organs (posterolateral view) **G** surstylus **H** phallic organs (ventral view) **I** phallic organs (dorsolateral view) **J** oviscapt (lateral view) **K** oviscapt (ventral view) **L** spermatheca. Scale bars: 1.0 mm (photograph) or 0.1 mm (line drawing).

This species is characteristic in sharing the spotted wings (Fig. 5C) with three known species of *Dettopsomyia*, *De.formosa* (Lamb 1914: fig. 33; Hardy 1965: fig. 20b), *De.jacobsoni* (Duda 1926: fig. 3) and *De.preciosa* (de Meijere 1911: fig. 49; Duda 1924: fig. 39), but differs in CS-code (AbCD??GHiJK?M) from them: *De.formosa* (ABCDEFGHiJKlM), *De.jacobsoni* (ABCDEFGHIJKLM) and *De.preciosa* (ABCDefGHIJKLm) (Table 3).

##### Description.

(♂, ♀; not repeating characters common to *De.acutipenis* sp. nov.). ***Head*** (Fig. 5A, B): Ocellar setae located just outside triangle made by ocelli. Frons yellow, with blackish brown stripes. Face gray. First flagellomere black.

***Thorax*** (Fig. 5A, B): Scutum, scutellum, and thoracic pleura yellow. Scutum with confluent stripes. Scutellum with somewhat H-shaped, dark marking medially and dark stripes covering bases of ipsilateral scutellar setae laterally. Acrostichal setulae in 2–4 vestigial rows. Dorsocentral setae two pairs.

***Wing*** (Fig. 5C) grayish yellow, black to blackish brown basally, with four dark spots along anterior margin and 14 scattered, pale spots. Veins brown. R_2+3_ waved, strongly curved to costa apically; R_4+5_ and M_1_ distally diverged from each other.

***Legs*** (Fig. 5A) pale brown.

***Abdomen*** (Fig. 5D, E): Tergites blackish brown, laterally with yellowish spots: one per side on tergite II, two per side on tergites III–VI.

***Male terminalia*** (Fig. 5F–I): Epandrium with three setae per side laterally; ventral lobe short, narrow, apically round and sclerotized like peg, with two small setae subapically. Surstylus with prensisetae arranged in V-shape (approximately seven on caudal margin and 6–8 in oblique row on outer surface; one or two dorsalmost on outer surface somewhat separated from others), several upward-curved setae on ventral to subventral portion of inner surface and one or two trichoid setae on outer surface near base of epandrial ventral lobe. Cercus broadly fused to epandrium, pubescent anteriorly, with approximately 28 setae; several setae along caudoventral margin shorter. Hypandrium somewhat trapezoid; apodeme anteriorly truncate, twice as wide as long. Paramere fused to hypandrium, not pubescent but with a single setula. Aedeagus distally membranous, subapically dilated and serrated on lateral margins around gonopore, apically sharply pointed; apodeme as long as aedeagus.

***Female terminalia*** (Fig. 5J–L): Oviscapt with one trichoid and two peg-like lateral ovisensilla and 15 marginal peg-like ovisensilla more loosely arranged anteriorly; distal portion approximately 1/4 of whole length, convex on dorsal margin in lateral view. Spermathecal capsule as broad as long, finely wrinkled on basal half, with shallow apical indentation; introvert ca. 4/5 height of outer capsule.

***Measurements***: BL = 1.51 mm in holotype (range in 3♂ paratypes: 1.41–1.66 mm; range in 3♀ paratype: 1.53–1.80 mm); ThL = 0.57 (0.57; 0.58–0.62) mm; WL = 1.37 (1.34–1.41; 1.47–1.52) mm; WW = 0.73 (0.66–0.71; 0.71–0.74) mm.

***Indices***: arb = 3/2 (range in 3♂, 3♀, or less if noted, paratypes: 3 or 4/2), FW/HW = 0.60 (0.59–0.62), ch/o = 0.38 (0.33–0.47), prorb = 0.60 (0.49–0.70), rcorb = 0.20 (0.20–0.23), vb = 0.35 (0.34–0.67), dcl (anterior dorsocentral seta/posterior dorsocentral seta in length) = 0.74 (0.82–0.89), sctl = (n/a)/(1♂: 0.90), sterno = 0.71 (0.44–0.68), orbito = 0.29 (0.21–0.26), dcp (distance between ipsilateral dorsocentral setae/distance between anterior dorsocentral setae) = 0.71 (0.65–0.81), sctlp = 1.27 (1.03), C = 1.00 (0.89–1.16), 4c = 2.51 (1.71–2.79), 4v = 2.24 (1.79–2.82), 5× = 1.90 (1.68–2.01), ac = 2.78 (2.13–3.05), M = 0.87 (0.84–1.10), C3F = 0.52 (0.54–0.69).

##### Distribution.

China (Yunnan).

##### Etymology.

Referring to the serrated, lateral margins of the gonopore of the aedeagus.

#### Dettopsomyia discontinua

Taxon classificationAnimaliaDipteraDrosophilidae

Wang & Gao sp. nov.

7FBA78AF-48AB-51D8-8478-90C3500217B3

http://zoobank.org/A21C488D-0012-4CB4-9485-93577A0B454E

Figure 6

##### Material.

***Holotype***: ♂ (#01585), Banpo, Yixiang, Simao, Pu’er, Yunnan, China, ca. 1300 m (22°44'N, 101°.07'E), by net sweeping above herbs, 2.x.2012 (J.J. Gao) (KIZ). ***Paratypes***: China: 1♂ (#01584), same data as holotype; 5♂, 1♀ (#01139–1144), Zaotanghe, Baihualing, Baoshan, Yunnan, ca. 1540 m (25°18'N, 98°47'E), 4.viii.2012, *ex* small mushroom (J.J. Gao); 3♀, 3♂ (#01167–1169, #01172–1174), from decaying aroid (*Rhaphidophoradecursiva*) infructescences collected from Baihualing, Baoshan, Yunnan, 23.ix.2012 (J.J. Gao, Z. Fu, and J.M. Chen) (KIZ, SEHU).

##### Diagnosis.

**Figure 6.**
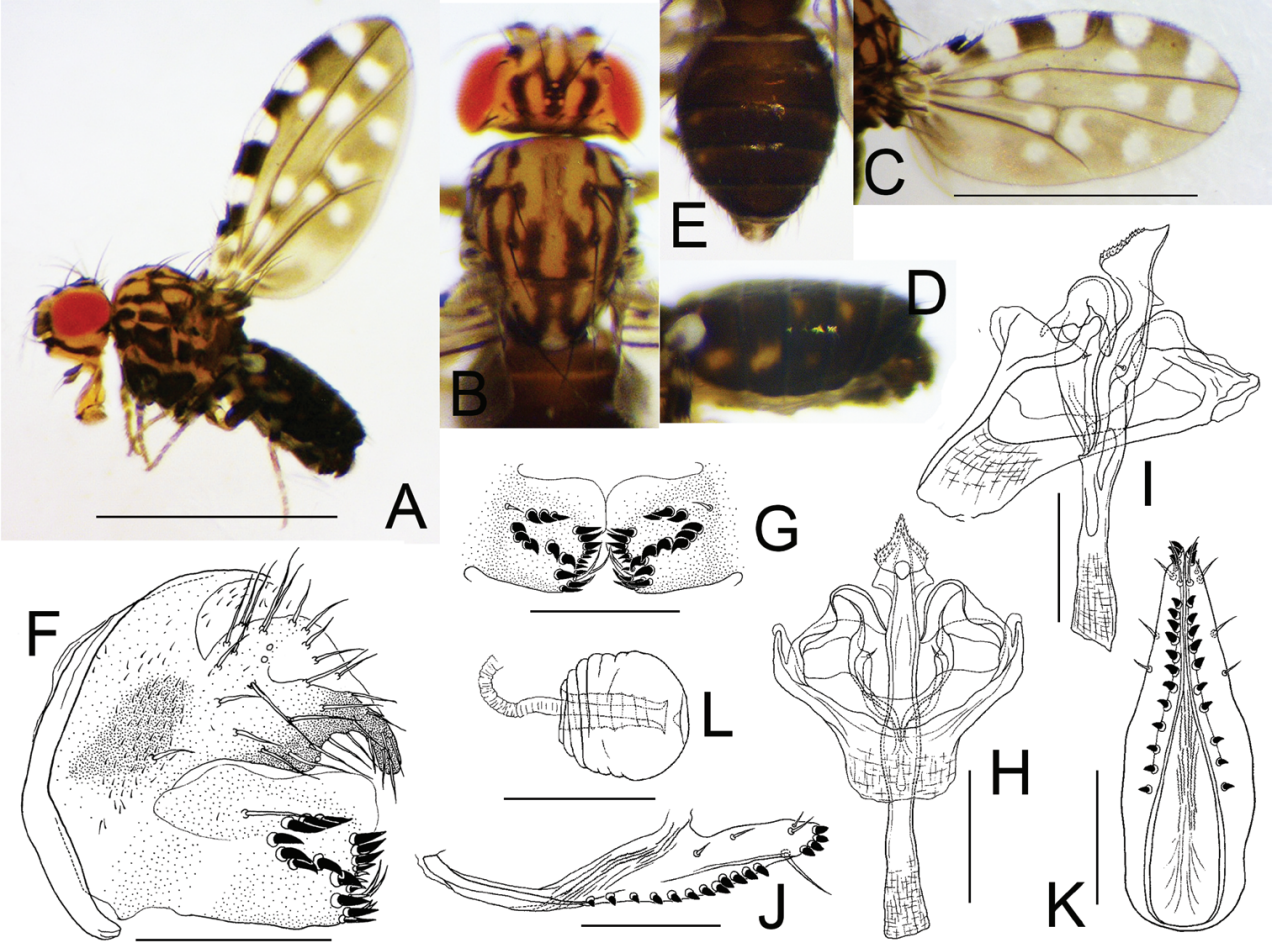
*Dettopsomyiadiscontinua* Wang & Gao, sp. nov. (**A–I** #01585, **J–L** paratype #01168) **A** left lateral habitus **B** head and thorax (dorsal view) **C** wing (right, dorsal view) **D** abdomen (lateral view) **E** abdomen (dorsal view) **F** periphallic organs (posterolateral view) **G** surstylus **H** phallic organs (ventral view) **I** phallic organs (dorsolateral view) **J** oviscapt (lateral view) **K** oviscapt (ventral view) **L** spermatheca. Scale bars: 1.0 mm (photograph) or 0.1 mm (line drawing).

This species is closely related to *De.serripenis* sp. nov., forming a highly supported (BP = 100) clade with it (Fig. 1). These two species are indistinguishable in CS-code from each other: *De.discontinua* sp. nov. (AbCD?FGHiJKLM) and *De.serripenis* sp. nov. (AbCD??GHiJK?M). However, they can be easily distinguished from each other by the following characters: 1) cercus caudoventrally strongly sclerotized and protruded ventrad like finger (Fig. 6F) in *De.discontinua* sp. nov. (abbreviated *Dd* here), but only pointed at caudoventral corner (Fig. 5F) in *De.serripenis* sp. nov. (abbreviated *Ds*); 2) surstylus with approximately 11 prensisetae on distal margin and nine or ten ones on medial portion of outer surface, arranged together nearly in circle (Fig. 6F, G) in *Dd*, but with 14 or 15 prensisetae arranged in V-shape (Fig. 5F, G) in *Ds*; and 3) marginal peg-like ovisensilla in row interrupted around subterminal, long, trichoid seta (Fig. 6J, K) in *Dd*, but in continuous row (Fig. 5J, K) in *Ds*.

##### Description.

(♂, ♀; not repeating characters common to *De.serripenis* sp. nov.). ***Head*** (Fig. 6A, B): Frons with black stripes. Gena yellow. Palpus grayish yellow.

***Thorax*** (Fig. 6A, B): Scutum, scutellum, and thoracic pleura with color patterns similar to those of *De.serripenis* sp. nov. Acrostichal setulae in two rows. Basal scutellar setae slightly converged.

***Wing*** (Fig. 6C): Wing maculated as in *De.serripenis* sp. nov.

***Legs*** (Fig. 6A) pale grayish yellow.

***Abdomen*** (Fig. 6D, E): Tergites blackish brown to black; II–V each laterally with a pale brown spot per side.

***Male terminalia*** (Fig. 6F–I): Epandrium pubescent on mediolateral portion only, with one seta per side on mediolateral portion; ventral lobe not differentiated; apodeme narrow, somewhat triangular. Surstylus somewhat quadrate, large plate, with one trichoid seta and 10–11 prensisetae in sinuated row on outer surface and 6–8 prensisetae decreasing in size downward on caudal margin. Cercus unpubescent, with approximately 33 setae. Hypandrium somewhat hemicircular; apodeme slightly wider than long. Aedeagus subapically with a pair of triangular lateral flaps; apodeme shorter than aedeagus.

***Female terminalia*** (Fig. 6J–L): Oviscapt with three trichoid lateral ovisensilla, 14–16 peg-like marginal ovisensilla and one subterminal, trichoid, long seta; distal portion approximately 1/3 of whole length, nearly flat on dorsal margin in lateral view.

***Measurements***: BL = 1.50 mm in holotype (range in 5♂ paratypes: 1.42–1.67 mm; range in 4♀ paratypes: 1.57–1.75 mm); ThL = 0.52 (0.55–0.64; 0.55–0.68) mm; WL = 1.30 (1.26–1.37; 1.36–1.58) mm; WW = 0.66 (0.62–0.70; 0.63–0.80) mm.

***Indices***: arb = 4 or 5/2 (range in 5♂, 4♀, or less if noted, paratypes: 4/2), FW/HW = 0.60 (0.57–0.61), ch/o = 0.38 (0.35–0.49), prorb = 0.72 (0.59–0.77), rcorb = 0.14 (0.16–0.21), vb = 0.32 (0.22–0.46), dcl = 0.74 (0.72–0.85), sctl = 0.98 (0.95–1.12), sterno = 0.73 (0.44–0.64), orbito = 0.19 (0.25–0.32), dcp = 0.65 (0.65–1.08), sctlp = 1.06 (0.95–1.14), C = 0.88 (0.90–0.96), 4c = 2.53 (2.11–2.34), 4v = 2.39 (1.93–2.30), 5× = 2.04 (1.63–2.09), ac = 3.18 (2.68–3.06), M = 0.93 (0.78–0.85), C3F = 0.58 (0.42–0.63).

##### Distribution.

China (Yunnan).

##### Etymology.

Referring to the interruptedly arranged marginal ovisensilla.

#### Dettopsomyia camelonota

Taxon classificationAnimaliaDipteraDrosophilidae

Wang, Li & Gao sp. nov.

28019CDA-45F9-5928-A638-FBFC2E7CFBCF

http://zoobank.org/7BCD32DA-ABD3-47B3-AAC9-5DF315757B63

Figure 7

##### Material.

***Holotype***: ♂ (#01607), Banpo, Yixiang, Simao, Pu’er, Yunnan, China, ca. 1300 m (22.73°N, 101.12°E), 25.x.2012 (J.J. Gao) (KIZ). ***Paratype***: China: 1♀ (#01608), same data as holotype (KIZ).

##### Diagnosis.

**Figure 7.**
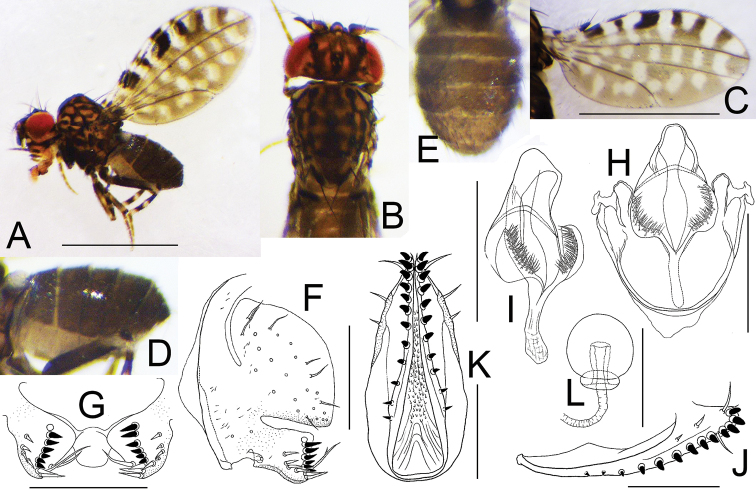
*Dettopsomyiacamelonota* Wang, Li & Gao, sp. nov. (**A–I** #01607, **J–L** paratype #01608) **A** left lateral habitus **B** head and thorax (dorsal view) **C** wing (right, dorsal view) **D** abdomen (lateral view) **E** abdomen (dorsal view) **F** periphallic organs (posterolateral view) **G** surstylus **H** phallic organs (ventral view) **I** phallic organs (dorsolateral view) **J** oviscapt (lateral view) **K** oviscapt (ventral view) **L** spermatheca. Scale bars: 1.0 mm (photograph) or 0.1 mm (line drawing).

This species differs from all the remaining congeneric species in CS-code (ABCD?FGhiJK?m) (Table 3) and having the following diagnostic characters: unique, distinctly humpbacked scutum in lateral view (Fig. 7A); aedeagus characterized by large, vault-like arch (Fig. 7H, I); and large, spoon-shaped paramere densely hirsute on inner surface (Fig. 7H, I). It resembles *De.formosa*, *De.jacobsoni*, *De.serripenis* sp. nov., and *De.discontinua* sp. nov. in wing marking pattern, but can be distinguished from them by much more (approximately 24) pale spots (Fig. 7C).

##### Description.

(♂, ♀; not repeating characters common to *De.acutipenis* sp. nov.). ***Head*** (Fig. 7A, B): Ocellar setae located just inside triangle made by ocelli. Frons with blackish brown stripes. Face grayish yellow to blackish brown; carina broad, dorsally strongly swollen and blackish brown, medially yellowish brown, ventrally nearly flat, black and with broad, pale yellow, traverse band. Clypeus blackish yellow. Gena pale yellow, ventrally black. Palpus gray, paddle-shaped in ventral view, with one prominent apical seta and several ventral ones. Antennal pedicel long triangular, black, laterally with yellowish patch; 1^st^ flagellomere long, somewhat triangular, black, with pale patch on inner, dorsal margin; arista with brown dorsal and ventral branches nearly as long as whitish trunk.

***Thorax*** (Fig. 7A, B): Scutum, scutellum, and thoracic pleura grayish yellow. Scutum with blackish brown to black, longitudinal stripes interweaved with each other. Scutellum medially with blackish brown to black patch merged with lateral black spots covering bases of ipsilateral scutellar setae. Acrostichal setulae in two vestigial rows. Dorsocentral setae three pairs; anteriormost pair distinctly shorter and thinner, located slightly anterior to transverse suture and more widely separated from each other. Basal scutellar setae divergent.

***Wing*** (Fig. 7C): Veins brown. R_2+3_ not waved medially, strongly curved to costa apically. R_4+5_ and M_1_ veins distally nearly parallel with each other.

***Legs*** (Fig. 7A) yellow to pale brown.

***Abdomen*** (Fig. 7D, E): Tergites blackish brown.

***Male terminalia*** (Fig. 7F–I): Epandrium nearly completely smooth, with two setae per side on ventral portion; ventral lobe very small; somewhat triangular apodeme present on anteromedial margin. Surstylus with a row of approximately six prensisetae on distal margin, approximately four thick, short setae on submedial to ventral portion of outer surface and a few trichoid setae around caudoventral corner. Cercus broadly fused to epandrium, large, somewhat fan-shaped, caudoventrally not pointed, nearly smooth, with approximately 25 short setae. Hypandrium broad, anteriorly rounded, with triangular apodeme. Aedeagus bilobed; apodeme approximately 1/2 as long as aedeagus.

***Female terminalia*** (Fig. 7J–L): Oviscapt with three lateral trichoid and 12 or 13 marginal, apically somewhat blunt peg-like ovisensilla. Spermathecal capsule spherical, strongly constricted near base; introvert ca. 3/5 height of outer capsule.

***Measurements***: BL = 1.54 mm in holotype (1♀ paratype: 1.70 mm); ThL = 0.57 (0.65) mm; WL = 1.42 (1.53) mm; WW = 0.72 (0.72) mm.

***Indices***: arb = 4/2 (1♀: 4/2), FW/HW = 0.65 (0.64), ch/o = 0.41 (0.48), prorb = 0.71 (n/a), rcorb = 0.18 (0.15), vb = 0.34 (0.29), dc_1_l = 0.36 (0.28), dc_2_l = n/a (0.72), sctl = 0.94 (0.95), sterno = n/a (0.80), orbito = 0.18 (0.21), dc_1_p = 0.39 (0.32), dc_2_p = 0.67 (0.62), sctlp = 1.14 (1.00), C = 0.94 (0.90), 4c = 2.75 (2.60), 4v = 3.00 (2.73), 5× = 1.85 (1.63), ac = 4.21 (4.17), M = 1.04 (0.87), C3F = 0.34 (0.50).

##### Distribution.

China (Yunnan).

##### Etymology.

A combination of the Greek words *camelos* and *notos*, referring to the humped, camel-like notum.

#### Dettopsomyia paranigrovittata

Taxon classificationAnimaliaDipteraDrosophilidae

Wang, Li & Gao sp. nov.

3DAC2539-113B-530D-8AB3-9DF7C8543743

http://zoobank.org/B1789016-279E-4E0A-8817-FE2D1F394558

Figure 8

##### Materials.

***Holotype***: ♂ (#01145), *ex.* inflorescence of *Rh.decursiva*, Laomengzhai, Baihualing, Baoshan, Yunnan, China, ca. 1500 m (25°17'N, 98°48'E), 3.viii.2012 (J.J. Gao) (KIZ). ***Paratype***: China: 1♂ (#01579), emerged (together with many adults of *De.nigrovittata*) from decaying spathes of *Rh.decursiva* collected from Laomengzhai, Baihualing, Baoshan, Yunnan (same as holotype) and cultured in laboratory, 23.ix.2012 (J.J. Gao, Z. Fu, J.M. Chen) (KIZ).

##### Diagnosis.

**Figure 8.**
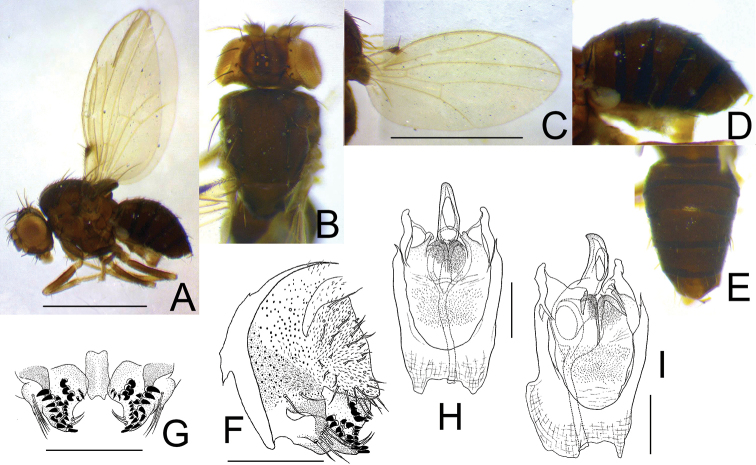
*Dettopsomyiaparanigrovittata* Wang, Li & Gao, sp. nov. (**A–I** #01145) **A** left lateral habitus **B** head and thorax (dorsal view) **C** wing (right, dorsal view) **D** abdomen (lateral view) **E** abdomen (dorsal view) **F** periphallic organs (posterolateral view) **G** surstylus **H** phallic organs (ventral view) **I** phallic organs (ventrolateral view). Scale bars: 1.0 mm (photograph) or 0.1 mm (line drawing).

This species closely resembles *De.nigrovittata* in the external morphology and male terminalia, but can be distinguished from it by the surstylus chaetotaxy: in *De.paranigrovittata* sp. nov., approximately 23 subequal, peg-like prensisetae arranged roughly in five sets on medial to distal portion of outer surface and two upward-curved, trichoid setae on subventral portion of inner surface (Fig. 8F, G); but in *De.nigrovittata*, approximately 25 more or less heteromorphic setae arranged in three rows on upper half of outer surface and two larger setae at lower tip (Okada 1956: fig. 31C, as *De.argentifrons*).

##### Description.

(♂; not repeating characters common to *De.acutipenis* sp. nov.). ***Head*** (Fig. 8A, B): Eye nearly rectangular to body axis. Fronto-orbital plate yellowish brown. Frons and frontal vittae grayish brown, somewhat shining. Face grayish yellow, black at middle and lateral sides; carina grayish yellow, rather prominent. Gena blackish brown, with pale spots antero- and medio-dorsally. Palpus grayish yellow, with large black spot. Antennal pedicel grayish yellow; 1^st^ flagellomere dark gray. Subvibrissal seta short.

***Thorax*** (Fig. 8A, B): Scutum blackish brown to black, with four narrow, silver stripes; scutellum and thoracic pleura blackish brown. Acrostichal setulae in six rows. Dorsocentral setae two pairs. Basal scutellar setae divergent.

***Wing*** (Fig. 8C) hyaline. Veins pale brown. R_2+3_ nearly straight; R_4+5_ and M_1_ distally parallel. Haltere pale whitish to grayish yellow.

***Legs*** (Fig. 8A) pale brown to blackish brown.

***Abdomen*** (Fig. 8D, E): Tergites entirely black.

***Male terminalia*** (Fig. 8F–I): Epandrium pubescent on lateral to dorsal portion, with one and two setae per side on sub-dorsal and -ventral portions, respectively; ventral lobe distally with five long, trichoid setae; apodeme on anterior margin. Surstylus somewhat triangular, large plate. Cercus broadly fused to epandrium, somewhat roundish at caudoventral corner, entirely pubescent, with approximately 36 setae. Hypandrium anteriorly slightly narrower, caudomedially notched. Paramere fused to hypandrium, densely pubescent, apically with one minute setula. Aedeagus curved ventrad proximally but dorsad distally, distally narrowing like horn in lateral view, ventro-subapically with large, oval gonopore; apodeme slightly shorter than aedeagus.

***Measurements***: BL = 1.69 mm in holotype (1♂ paratype: 1.52 mm); ThL = 0.68 (0.65) mm; WL = 1.50 (1.40) mm; WW = 0.74 (0.71) mm.

***Indices***: arb = 4/2 (1♂ paratype: 4/2), FW/HW = 0.56 (0.55), ch/o = 0.36 (0.41), prorb = 0.68 (0.73), rcorb = n/a (0.32), dcl = 0.71 (0.71), sctl = 0.83/(0.85), sterno = n/a (0.61), orbito = 0.57 (0.52), dcp = 0.80 (0.83), sctlp = 0.91 (0.92), C = 1.36 (1.23), 4c = 1.94 (2.20), 4v = 2.92 (3.15), 5× = 3.20 (2.67), ac = 4.73 (3.89), M = 1.23 (1.25), C3F = 0.60 (0.65).

##### Distribution.

China (Yunnan).

##### Etymology.

Referring to the close morphological affinity to *De.nigrovittata*.

## Discussion

Since the early days of taxonomy for *Dettopsomyia* and *Styloptera*, these two genera have been ambiguous in their systematic positions. Until now, only few phylogenetic studies have been conducted to clarify the relationships between them. Grimaldi (1990) classified these two genera with *Jeannelopsis* Seguy, *Tambourella* Wheeler, *Mulgravea* Bock, *Sphaerogastrella* Duda, *Hypselothyrea* de Meijere, and *Liodrosophila* Duda in the *Styloptera* genus group, based on a cladistic analysis using 217 characters of 120 species. However, each genus was represented by a single species in his analysis. In Yassin’s (2013) Bayesian phylogenetic tree based on DNA sequences of 70 genera of the Drosophilidae, *Styloptera* (represented by *S.formosae* only) was coupled with the subgenusDorsilopha Sturtevant (represented by *Drosophilabusckii* Coquillett, 1901), and *Dettopsomyia* (represented by *De.nigrovittata*) was placed into a clade containing the genera *Jeannelopsis*, *Dichaetophora* Duda, *Hirtodrosophila* Duda, *Zygothrica* Wiedemann, and *Mycodrosophila* Oldenberg. To completely solve this ambiguity in the systematics of the subfamily Drosophilidae a full-scale molecular phylogenetic analysis should be conducted with extensive taxon sampling from *Dettopsomyia*, *Styloptera* and putatively related genera and subgenera. The species diversity of *Dettopsomyia* and *Styloptera* has also been less explored: only 13 and ten species were known, respectively, before the present study. Our finding of five new *Dettopsomyia* species from southwestern China (Fig. 3) suggests that more unknown species remain to be discovered from the Oriental region. To precisely delimit the boundaries of these genera, as many species as possible need to be incorporated into the systematic study.

The natural history of *Dettopsomyia* flies is still less explored. However, our collection records suggest their florivorous nature. Adult flies of *De.acutipenis* sp. nov. and *De.serripenis* sp. nov. were collected from flowers of Zingerberaceae, *De.serripenis* sp. nov. also from inflorescences of *Alocasiaodora* (Araceae), and *De.paranigrovittata* from an inflorescence of *Rhaphidophoradecursiva* (Araceae). Additionally, offspring adults of *De.paranigrovittata* sp. nov., *De.discontinua* sp. nov., and *De.nigrovittata* emerged from infructescences with decayed spathe of *R.decursiva* in laboratory rearings, and Carson and Okada (1980) reported rearing *De.repletoides* (under the name of *Stylopterarepletoides*) from infructescences of *Colocasiaesculenta* (Araceae). Wheeler (1951) observed dipteran larvae and puparia in a rotting, bleeding part of banana plant, and adults emerged from them and were identified as *De.nigrovittata*. Thus, some species of *Dettopsomyia* use decayed plant materials as a breeding substrate.

## Supplementary Material

XML Treatment for Dettopsomyia

XML Treatment for Dettopsomyia acutipenis

XML Treatment for Dettopsomyia serripenis

XML Treatment for Dettopsomyia discontinua

XML Treatment for Dettopsomyia camelonota

XML Treatment for Dettopsomyia paranigrovittata
